# GenoMycAnalyzer: a web-based tool for species and drug resistance prediction for *Mycobacterium* genomes

**DOI:** 10.1186/s12864-024-10320-3

**Published:** 2024-04-20

**Authors:** Doyoung Kim, Jeong-Ih Shin, In Young Yoo, Sungjin Jo, Jiyon Chu, Woo Young Cho, Seung-Hun Shin, Yeun-Jun Chung, Yeon-Joon Park, Seung-Hyun Jung

**Affiliations:** 1 Department of Biomedicine & Health Sciences, College of Medicine, The Catholic University of Korea, Seoul, Korea; 2 Integrated Research Center for Genomic Polymorphism, Precision Medicine Research Center, College of Medicine, The Catholic University of Korea, Seoul, Korea; 3 Department of Laboratory Medicine, Seoul St. Mary’s Hospital, College of Medicine, The Catholic University of Korea, Seoul, Korea; 4 Department of Laboratory Medicine, Eunpyeong St. Mary’s Hospital, College of Medicine, The Catholic University of Korea, Seoul, Korea; 5 ConnectaGen, Hanam, Korea; 6 Departments of Microbiology, College of Medicine, The Catholic University of Korea, Seoul, Korea; 7 Departments of Biochemistry, College of Medicine, The Catholic University of Korea, 222 Banpo-daero, Seoch-Gu, Seoul, 06591 Republic of Korea

**Keywords:** *Mycobacterium tuberculosis* complex, Non-tuberculosis mycobacteria, Drug susceptibility testing, Mycobacteria species identification, Whole genome sequencing

## Abstract

**Background:**

Drug-resistant tuberculosis (TB) is a major threat to global public health. Whole-genome sequencing (WGS) is a useful tool for species identification and drug resistance prediction, and many clinical laboratories are transitioning to WGS as a routine diagnostic tool. However, user-friendly and high-confidence automated bioinformatics tools are needed to rapidly identify *M. tuberculosis* complex (MTBC) and non-tuberculous mycobacteria (NTM), detect drug resistance, and further guide treatment options.

**Results:**

We developed GenoMycAnalyzer, a web-based software that integrates functions for identifying MTBC and NTM species, lineage and spoligotype prediction, variant calling, annotation, drug-resistance determination, and data visualization. The accuracy of GenoMycAnalyzer for genotypic drug susceptibility testing (gDST) was evaluated using 5,473 MTBC isolates that underwent phenotypic DST (pDST). The GenoMycAnalyzer database was built to predict the gDST for 15 antituberculosis drugs using the World Health Organization mutational catalogue. Compared to pDST, the sensitivity of drug susceptibilities by the GenoMycAnalyzer for first-line drugs ranged from 95.9% for rifampicin (95% CI 94.8–96.7%) to 79.6% for pyrazinamide (95% CI 76.9–82.2%), whereas those for second-line drugs ranged from 98.2% for levofloxacin (95% CI 90.1–100.0%) to 74.9% for capreomycin (95% CI 69.3–80.0%). Notably, the integration of large deletions of the four resistance-conferring genes increased gDST sensitivity. The specificity of drug susceptibilities by the GenoMycAnalyzer ranged from 98.7% for amikacin (95% CI 97.8–99.3%) to 79.5% for ethionamide (95% CI 76.4–82.3%). The incorporated Kraken2 software identified 1,284 mycobacterial species with an accuracy of 98.8%. GenoMycAnalyzer also perfectly predicted lineages for 1,935 MTBC and spoligotypes for 54 MTBC.

**Conclusions:**

GenoMycAnalyzer offers both web-based and graphical user interfaces, which can help biologists with limited access to high-performance computing systems or limited bioinformatics skills. By streamlining the interpretation of WGS data, the GenoMycAnalyzer has the potential to significantly impact TB management and contribute to global efforts to combat this infectious disease. GenoMycAnalyzer is available at http://www.mycochase.org.

**Supplementary Information:**

The online version contains supplementary material available at 10.1186/s12864-024-10320-3.

## Background

The *Mycobacterium* genus comprises more than 190 species and subspecies, including the *Mycobacterium* tuberculosis complex (MTBC) and non-tuberculosis mycobacteria (NTM) [1]. Tuberculosis (TB), a bacterial infection caused by MTBC, is one of the leading causes of death worldwide. Millions of newly diagnosed TB cases and 1.6 million TB-related deaths were reported in 2021 [2]. The effective treatment and management of TB is challenging owing to the emergence of multidrug-resistant TB. Globally, the treatment success rate for patients newly diagnosed with TB is 86%, whereas that for patients diagnosed with multidrug-resistant TB is only 60% [2]. Thus, timely detection of drug resistance is crucial to guide treatment options and prevent further transmission [3].

In addition to MTBC, NTM has gained recognition as an important human pathogen, as its incidence and prevalence continue to increase worldwide [4, 5]. NTM can be broadly categorized into two groups (rapidly- and slow-growing mycobacteria), of which *Mycobacterium avium* complex (MAC) and *Mycobacterium abscessus* complex are the most important species frequently isolated from patients with NTM infection [5, 6]. MTBC and NTM share similar microbiological properties and can lead to infections with overlapping clinical symptoms. However, they exhibit distinct disease characteristics and respond to different treatment options, and their susceptibility patterns to antimicrobial drugs vary depending on the species causing the infection [4].

Culture-based methods have traditionally been used as the gold standard for bacterial identification and diagnosis of drug-resistant TB. However, they are labor-intensive, require specialized infrastructure, and can take weeks to months [7]. Although molecular-based methods, such as GeneXpert and line probe assays, are more rapid than culture-based methods, they only target the most common resistance-associated variants (RAV) for a limited number of drugs [7, 8]. Whole-genome sequencing (WGS) is a useful tool for species identification, drug resistance prediction, and transmission tracing [9, 10]. In particular, WGS not only allows for the screening of well-known RAVs but also presents opportunities to uncover novel genetic alterations for both new and repurposed drugs. For example, WGS identified *rplC* p.Cys154Arg as a dominant mutation in linezolid-resistant isolates [11]. Mutations in *mmpR5* (Rv0678), *atpE*, and *pepQ*, and those in *ddn*, *fbiA*, *fbiB*, *fbiC*, *fbiD*, and *fgd1* have also been reported as potential mechanisms underlying bedaquiline and delamanid resistance, respectively [12, 13]. More recently, the World Health Organization (WHO) published the first comprehensive catalogue of mutations in MTBC, applying rigorous classification criteria to assign a confidence grade to each variant associated with drug resistance [10].

Despite the many advantages of WGS, its application in routine clinical settings has been limited primarily owing to the requirement of bioinformatics expertise and high-performance computing systems [14]. In addition, the lack of expertise in command line bioinformatics among biologists has hinder the widespread use of WGS data. To overcome these challenges, it is necessary to develop analytical tools that meet the following functionalities: 1) identification of both MTBC and NTM at the species level; 2) prediction of drug-resistant TB based on an endorsed knowledge base, such as the WHO mutational catalogue; 3) user-friendliness and low system requirements, including graphical user interface (GUI) or web-based software, and 4) data quality control and visualization. Given that mutation-calling algorithms can yield inaccurate results depending on the quality of the read and mapping accuracy, visualizing and reviewing detected mutations before the final report is crucial [15]. Although several tools for analyzing mycobacterial genomes have been developed in recent years, including KvarQ, PhyResSE, Mykrobe, TBProfiler, ReSeqTB-UVP, and SAM-TB [16–21], software that integrates all these functionalities is scarce (Additional file 1: Table S1). Additionally, variations in drug resistance prediction results can occur between software packages owing to differences in the analysis pipelines and knowledge bases.

In this study, we developed GenoMycAnalyzer, a web-based software program that integrates functions for the identification of MTBC and NTM species and their lineages and spoligotype prediction, variant calling, annotation, drug resistance prediction, and visualization. The performance of the GenoMycAnalyzer pipeline was evaluated using publicly available WGS data.

## Implementation

### Quality control of raw sequence reads

Sequencing adapters and low-quality bases were trimmed using Cutadapt version 4.2 [22]. Sequencing reads with a Phred base quality score greater than 20 and lengths longer than 50 bp were retained. The base quality of the fastq files before and after trimming was assessed using FastQC version 0.11.9 [23]. To remove the sequencing reads originating from the host genome, the trimmed reads were aligned to the human reference genome (hg38) using BWA MEM version 0.7.17 [24]. The mapping status, including the total number of bases and reads, duplicate rate, mapping rate, and sequencing depth, was calculated using SAMtools version 1.16.1 [25], and only unmapped reads were extracted for downstream analyses.

### Species identification and spoligotyping

The identification of MTBC and NTM at the species level was performed using Kraken2 version 2.1.2, a taxonomic classification system based on *k*-mer matches [26]. To improve species predictions, a custom-built database containing 107 sequences from 18 NTM species in addition to the Kraken2 built-in database version 2023–8-18 was constructed (Additional file 1: Table S2). Subsequently, Braken software was used to estimate the abundance of species using taxonomy labels assigned by Kraken2 [26]. The most abundant species estimated by Braken was assigned to the species in the sample. Samples with fewer than 200 sequence reads mapped to the *Mycobacterium* genus or with a mapping rate of less than 10% to a predicted *Mycobacterium* species were excluded from further analyses. For MTBC, SpoTyping version 2.1 was used to determine the spoligotype from trimmed sequence reads [27]. The output is presented as an octal code.

### Variant calling and lineage prediction

For isolates identified as MTBC, sequencing reads were mapped to the H37Rv reference genome (GenBank accession number: NC_000962) using BWA MEM, as previously described [28]. Duplicate reads were marked and de-duplicated using SAMtools version 1.16.1 [25]. Single-nucleotide variants (SNVs) and small indels were identified using BCFtools version 1.16 with the ‘ploidy’ option set to 1 and the ‘consensus-caller’ model [29]. SnpEff version 5.1d was used to define mutations in genomic sequences and predict their functional consequences [30]. To obtain a reliable and robust mutation calling, the following variants were eliminated: (i) read depth < 30, (ii) Phred quality score < 30, (iii) mapping quality score < 30, and (iv) variant allele frequency (VAF) < 5%. Lineages were predicted based on a previously reported single-nucleotide polymorphisms (SNP) barcoding assay using an in-house Python script [31, 32].

### Detection of a large deletion

A large deletion profile at the gene level (> 50 bp) was estimated using the WGS data. The target genes were limited to four (*pncA*, *katG*, *gid*, and *ethA*) known to be associated with drug resistance [33]. Large deletions were defined as regions encompassing at least 50 bp with a sequencing depth of less than three standard deviations from the mean or less than ten mapped reads [34]. For comparison, large deletions were also predicted using the DELLY software version 1.1.7 with default parameters [35]. All identified large deletions were manually inspected using the Integrative Genomics Viewer (IGV) browser version 2.15.5 [15].

### Genotypic drug susceptibility testing

The annotated and filtered variants were classified into one of the following five groups according to the WHO mutational catalogue [10]: 1, associated with resistance; 2, associated with resistance-interim; 3, uncertain significance; 4, not associated with resistance-interim; and 5, not associated with resistance (neutral mutations). In brief, group 1 mutations were defined when the following five criteria were met: 1) a sum of resistant and susceptible isolates with a solo mutation ≥ 5, 2) a lower bound of the 95% confidence interval of the positive predictive value (PPV) conditional on being solo ≥ 25%, 3) odds ratio (OR) > 1, 4) OR of solo mutations > 1, and 5) false discovery rate-corrected *P* ≤ 0.05. Group 2 mutations were present as solo in *pncA* in at least two resistant isolates with a PPV of ≥ 50%, whereas group 4 mutations were present as solo in *pncA* with a PPV ≤ 40% and an upper bound of the 95% confidence interval of ≤ 75%. Group 3 comprised mutations that did not meet the criteria for inclusion in groups 1, 2, 4, or 5. We defined genotypic resistance to 15 antituberculosis drugs (isoniazid, rifampicin, ethambutol, pyrazinamide, levofloxacin, moxifloxacin, linezolid, bedaquiline, clofazimine, delamanid, amikacin, capreomycin, kanamycin, streptomycin, and ethionamide) as SNPs or indels overlapped with group 1 or 2 variants of the WHO mutational catalogue at either the nucleotide or amino acid level (Additional file 1: Table S3). In addition, large deletions of *pncA*, *katG*, *gid*, and *ethA* were considered resistant to pyrazinamide, isoniazid, streptomycin, and ethionamide, respectively. Variants falling into groups 3, 4, and 5 in the WHO mutational catalogue or mutations that did not meet the criteria outlined in the WHO mutational catalogue were considered genotypically susceptible. Detected RAVs were visualized in a Circos plot with the sequencing depth of the corresponding gene [36].

### Statistical analysis

The sensitivity, specificity, positive predictive value (PPV), and negative predictive value (NPV) were determined. The receiver operating characteristic (ROC) curve and area under the curve (AUC) were used to compare the predictive values of the WHO mutational catalogue and GenoMycAnalyzer datasets. All statistical analyses were performed using SPSS version 29 (IBM Corp., Armonk, NY, USA).

### Dataset collection and availability of the GenoMycAnalyzer

The inclusion criteria encompassed *Mycobacterium* spp. meeting the following conditions: isolates not used in the training of the WHO mutational catalogue, isolates with sequencing depth of 100 or more, and isolates with species information or pDST results. The pDST measurement method was not considered. According to these criteria, we collected a dataset of 8,259 Illumina raw sequence reads for which laboratory-based metadata were available from public datasets. Raw sequence data were downloaded from the European Nucleotide Archive (ENA), Sequence Read Archive (SRA), or DDBJ Sequence Read Archive. Among the datasets, those of 1,284 isolates were used to evaluate the accuracy of species identification, and those of 5,473 isolates with pDST results for at least one antituberculosis drug were used to evaluate the performance in predicting drug resistance. In addition, the datasets of 1,935 and 54 isolates were used to evaluate the accuracy of lineage and spoligotype predictions, respectively. Therefore, the data used for validation of each functionality comprise different datasets. Accession numbers and corresponding metadata are listed in Additional file 2: Table S4.

The GenoMycAnalyzer is accessible at https://www.mycochase.org/. This software is compatible with Windows, macOS, and Linux operating systems and is released under the GNU General Public License 3.0. The manual for the software can be found via the “help” tab on the GenoMycAnalyzer website and is accessible without the need to register or log in. Additionally, the source code for the data processing pipeline is available in the GitHub repository at https://github.com/IRCGP-Lab/GenoMycAnalyzer_Source.

### Data encryption and privacy

The GenoMycAnalyzer server encrypts all transmissions using the Fernet module from the cryptography package (https://github.com/pyca/cryptography). Fernet employs the Advanced Encryption Standard (AEC) algorithm for encryption, ensuring that encrypted data cannot be accessed without the key. This provides users with robust, secure communication with the web server.

## Results

### Pipeline overview

**Fig. 1:**
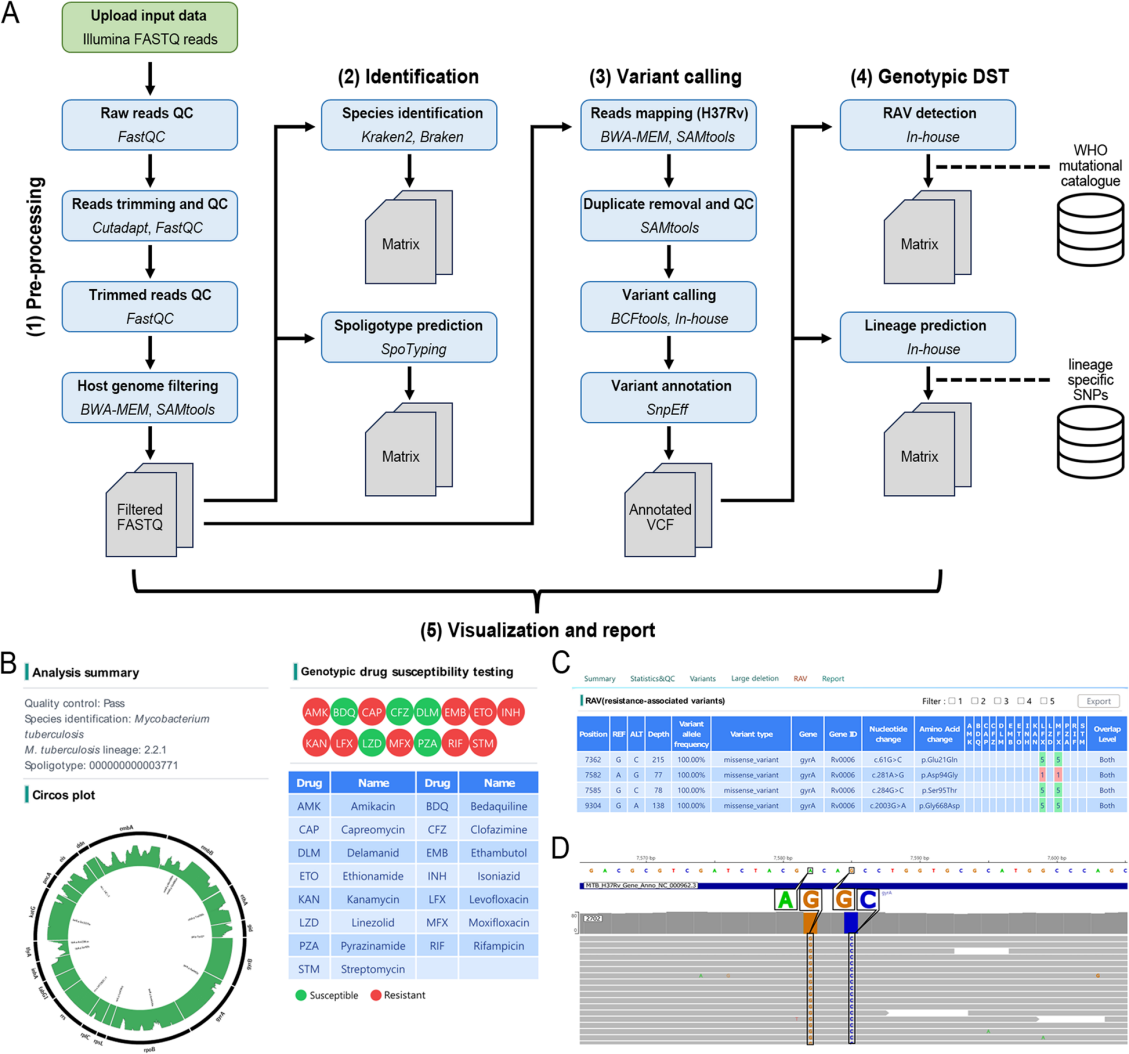
GenoMycAnalyzer pipelines. **A** The GenoMycAnalyzer platform includes five modules: pre-processing, identification, variant calling, genotypic DST, and visualization and report. (B-D) Example of WGS analyzed by GenoMycAnalyzer. **B** Partial result of the 'Analysis Summary' is displayed. The strain is identified as *M. tuberculosis*, with lineage (2.2.1) and spoligotype (000000000003771). Genotypic DST for 15 types of antimicrobial drugs is color-coded (resistant: red, susceptible: green). A Circos plot represents the sequencing coverage depth and detected variants for 17 genes harboring group 1 or 2 mutations. **C** Among the detected variants, overlapped variants with the WHO mutational catalogue are shown in ‘RAV (resistance-associated variants)’. **D** The reads mapping status of *gyrA* p.D94G, which confers resistance to fluoroquinolone, is shown in IGV implemented in GenoMycAnalyzer

GenoMycAnalyzer is a freely available web-based pipeline for users to analyze *Mycobacterium* genomes. The unique features of the GenoMycAnalyzer are listed in Table 1. The GenoMycAnalyzer consists of five sequential steps: 1) pre-processing and quality control, 2) species identification and molecular typing, 3) variant calling and annotation, 4) gDST, and 5) visualization and report generation (Fig. 1). GenoMycAnalyzer ingests single-end or paired-end fastq files generated by Illumina instruments as input, and supports the multiple file uploads and batch analyses. In the pre-processing step, GenoMycAnalyzer removes sequencing adaptors, low-quality bases, and sequencing reads originating from the host genome. Subsequently, the filtered reads are classified to the species level using a custom-built database that includes both MTBC and NTM sequences. If the isolate is predicted to be MTBC, GenoMycAnalyzer is used to perform subsequent analyses, such as lineage and spoligotype prediction, variant calling, annotation, and RAV identification. Notably, detected variants can be interactively inspected using the IGV browser [15], which helps determine the false-positive or false-negative calls. Finally, the analysis results are summarized in a report and downloaded in PDF file format. The run time for a sample with a sequencing depth of 300-fold is approximately 15 min. The uploaded data and processed files are retained on the server for three months. The GenoMycAnalyzer is available at http://www.mycochase.org.

**Table 1:** Features of GenoMycAnalyzer

Feature	Description
Quality control	● Provide various quality control statistics, including sequencing depth and mapping status
Identification	● Identify MTBC and NTM at species levels based on Kraken2 ● Provide improved species prediction based on a custom-built database
Molecular typing	● Provide lineage prediction based on genome-wide SNPs ● Provide spoligotype based on the direct repeat locus
Genotypic DST	● Provide gDST results for 15 antituberculosis drugs using the WHO mutational catalogue ● Detection of large deletions associated with drug resistance
Visualization	● Detected RAVs are visualized in a Circos plot, along with the sequencing depth of the corresponding gene ● Integrate the IGV browser to visualize and review the detected variants
Report	● Provide pdf formatted report with sample details, assay details, and genomic characteristics ● Users can edit additional comment in the report
Flexibility	● Users can modify the analysis parameters ● Users can add newly discovered RAVs to the knowledge database

### SNP-based genotypic DST performance of GenoMycAnalyzer

The GenoMycAnalyzer knowledge database comprises the 13,221 unique mutations reported in the WHO mutational catalogue, including 1,149 group 1 and 2 mutations known to confer drug resistance [10]. To evaluate the predictive performance of the GenoMycAnalyzer for gDST, we analyzed 5,473 MTBC genomes with pDST results for at least one antituberculosis drug. All isolates used in this evaluation were independent datasets not included in the training data of the WHO mutational catalogue. The mean coverage of the sequencing depth was 193.6x, with an average of 97.6% of bases covered by at least 50 reads in each isolate. The completeness of the pDST results differed depending on the drug, with the highest rates observed for the first-line drugs, rifampicin (94.4%), isoniazid (94.2%), ethambutol (84.0%), and pyrazinamide (60.4%) (Additional file 2: Table S4). The pDST data for second-line drugs ranged from 59.6% (streptomycin) to 1.9% (levofloxacin). Among the 5,473 isolates, 3,094 (56.5%) were pan-susceptible, 1,599 (29.2%) were multidrug-resistant TB (MDR-TB), and 77 (1.4%) were extensively drug-resistant TB (XDR-TB). The remaining 703 isolates (12.8%) were resistant to at least one drug. Four drugs were excluded from the evaluation because of the lack of RAVs (bedaquiline and clofazimine) or phenotypically resistant isolates (linezolid and delamanid).

**Fig. 2:**
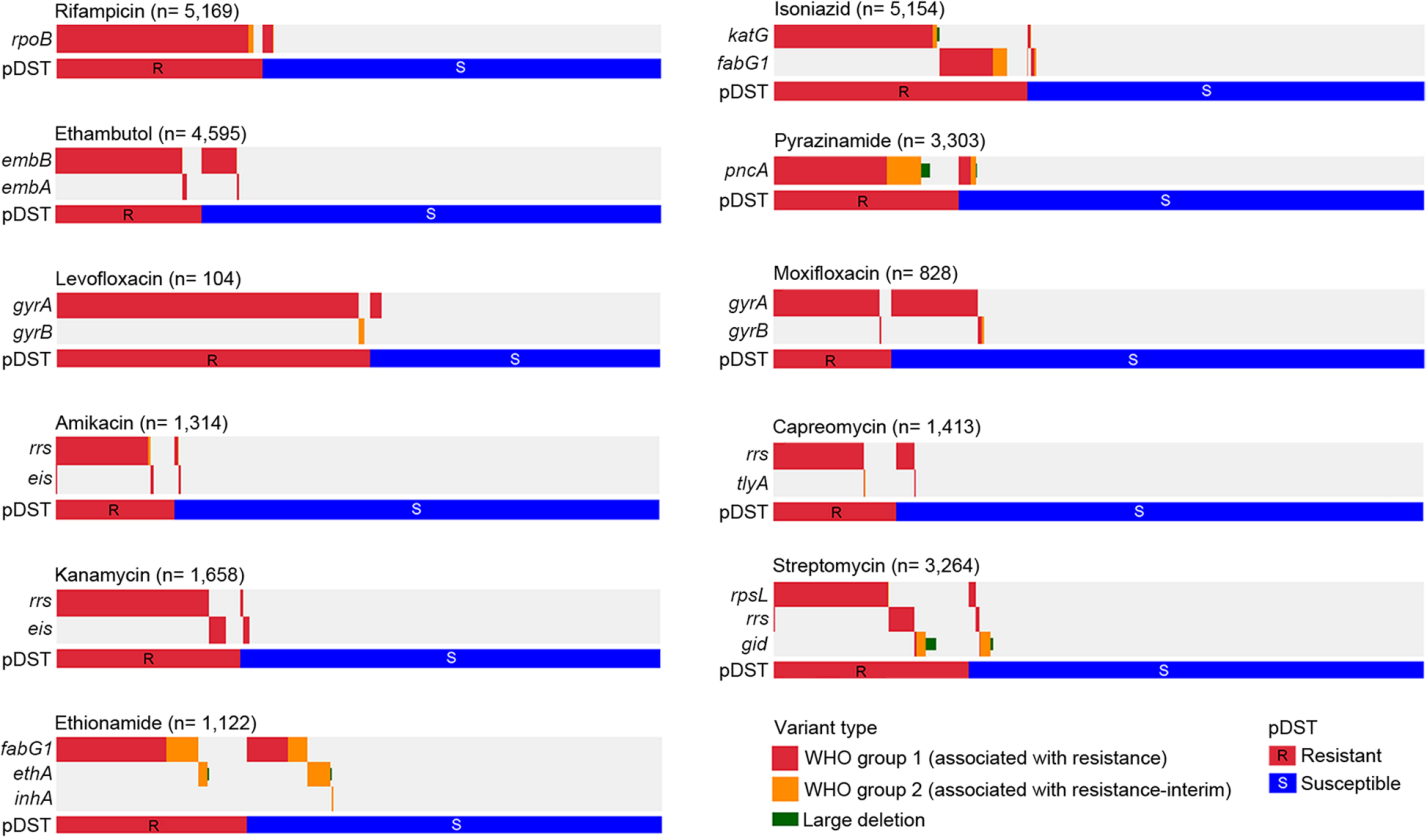
Genomic variants associated with 11 antimicrobial drugs. Genomic alterations and phenotypic DST to rifampicin, isoniazid, ethambutol, pyrazinamide, levofloxacin, moxifloxacin, amikacin, capreomycin, kanamycin, streptomycin, and ethionamide are shown. Each row and column represents the resistance-associated gene and strain, respectively

Overall, the GenoMycAnalyzer gDST for first-line drugs exhibited excellent predictive values (Fig. 2 and Table 2): 95.9% sensitivity and 97.3% specificity for rifampicin, 91.0% sensitivity and 97.7% specificity for isoniazid, and 89.9% sensitivity and 91.9% specificity for ethambutol. The sensitivity for pyrazinamide was lower than that for other first-line drugs (79.6% sensitivity and 96.2% specificity), which is consistent with a previous report [10]. The predictive values for second-line drugs varied for each drug, with sensitivities ranging from 98.2% (levofloxacin) to 74.9% (capreomycin) and specificities ranging from 98.7% (amikacin) to 79.5% (ethionamide) (Fig. 2 and Table 2). The sensitivity for assigning MDR-TB and XDR-TB was 88.2% (95% CI 86.5–89.7%) and 83.1% (95% CI 72.9–90.7%), respectively.

**Table 2:** Performances of GenoMycAnalyzer for genotypic drug susceptibility testing

Drug	Phenotypically resistant			Phenotypically susceptible			Performances^b^			
	**RAV**^**a**^		**Total**	**RAV**^**a**^		**Total**	**Sensitivity** **(95% CI)**	**Specificity** **(95% CI)**	**PPV** **(95% CI)**	**NPV** **(95% CI)**
	**Present**	**Absent**		**Present**	**Absent**					
Rifampicin	1688	73	1761	93	3315	3408	95.9% (94.8%-96.7%)	97.3% (96.7%-97.8%)	94.8% (93.7%-95.7%)	97.9% (97.3%-98.3%)
Isoniazid	1827	181	2008	71	3075	3146	91.0% (89.7%-92.2%)	97.7% (97.2%-98.2%)	96.3% (95.3%-97.0%)	94.4% (93.7%-95.1%)
Ethambutol	998	112	1110	284	3201	3485	89.9% (88.0%-91.6%)	91.9% (90.9%-92.7%)	77.9% (75.8%-79.7%)	96.6% (96.0%-97.2%)
Pyrazinamide	747	191	938	89	2276	2365	79.6% (76.9%-82.2%)	96.2% (95.4%-97.0%)	89.4% (87.2%-91.2%)	92.3% (91.3%-93.1%)
Levofloxacin	53	1	54	2	48	50	98.2% (90.1%-100.0%)	96.0% (86.3%-99.5%)	96.4% (87.2%-99.0%)	98.0% (87.3%-99.7%)
Moxifloxacin	137	13	150	118	560	678	91.3% (85.6%-95.3%)	82.6% (79.5%-85.4%)	53.7% (49.5%-58.0%)	97.7% (96.2%-98.6%)
Amikacin	213	45	258	14	1042	1056	82.6% (77.4%-87.0%)	98.7% (97.8%-99.3%)	93.8% (90.0%-96.3%)	95.9% (94.7%-96.8%)
Capreomycin	200	67	267	42	1104	1146	74.9% (69.3%-80.0%)	96.3% (95.1%-97.4%)	82.6% (77.8%-86.6%)	94.3% (93.1%-95.3%)
Kanamycin	465	39	504	25	1129	1154	92.3% (89.6%-94.4%)	97.8% (96.8%-98.6%)	94.9% (92.7%-96.5%)	96.7% (95.5%-97.5%)
Streptomycin	762	216	978	110	2176	2286	77.9% (75.2%-80.5%)	95.2% (94.2%-96.0%)	87.4% (85.2%-89.3%)	91.0% (90.0%-91.9%)
Ethionamide	280	73	353	158	611	769	79.3% (74.7%-83.4%)	79.5% (76.4%-82.3%)	63.9% (60.4%-67.3%)	89.3% (87.2%-91.2%)

^a^Present is the number of isolates with resistance-associated variants that overlap with high-confidence variants (grade 1 or 2) of the WHO mutational catalogue

*RAV* resistance-associated variant

^b^To calculate the predictive performances of GenoMycAnalyzer, pDST was assumed to be the gold standard

*PPV* positive predictive value, *NPV* negative predictive value

Next, we calculated the differences in AUC between the GenoMycAnalyzer and WHO mutational catalogue datasets and found that the predictive values for pyrazinamide, levofloxacin, and kanamycin were significantly better than those reported in the WHO mutational catalogue (Additional file 1: Table S5). In contrast, the predictive values for streptomycin and ethionamide were poorer than those of the WHO mutational catalogue. The predictive values for the remaining six drugs were not significantly different from those reported in the WHO mutational catalogue, suggesting the non-inferiority of the GenoMycAnalyzer (Additional file 1: Table S5).

### Discordance analysis in resistance predictions

To gain insights into the discrepancies between pDST and gDST, we examined RAVs for rifampicin and isoniazid, for which genotypic resistance mechanisms are well understood. To this end, we further analyzed isolates for which GenoMycAnalyzer predicted resistance while pDST was reported as susceptible (false positive; FP), and vice versa (false negative; FN) using TBProfiler (Additional file 3: Table S6) [19]. GenoMycAnalyzer called 74 FP predictions for isoniazid, and all of them had RAVs corresponding to group 1 or 2 mutations: *katG* p.S315T (*n* = 24), *fabG1* c.-15C > T (*n* = 22), *fabG1* c.-8 T > C (*n* = 14), *fabG1* p.L203L (*n* = 7), *fabG1* c.-8 T > A (*n* = 3), *katG* p.P429fs (*n* = 2), *katG* p.199 fs (*n* = 1), and *katG* p.Q471* (*n* = 1). Notably, all variants were consistently detected using TBProfiler except for two isolates. Similarly, all 101 FP predictions for rifampicin by GenoMycAnalyzer had RAVs corresponding to group 1 or 2 mutations, of which 88 (87.1%) were concordant with those of TBProfiler. The most frequent RAV for rifampicin FP predictions were *rpoB* p.S450L (*n* = 26) and p.I491F (*n* = 26), followed by p.L452P (*n* = 12), p.L430P (*n* = 8), and p.H445N (*n* = 5).

Among the 208 FN predictions for isoniazid, 130 isolates (62.5%) were identified as resistant, whereas the remaining 78 isolates (37.5%) were consistently predicted as susceptible (false negative) by TBProfiler (Additional file 3: Table S6). Notably, most variants detected in isolates predicted to be resistant by TBProfiler were also detected using GenoMycAnalyzer (*n* = 105, 80.8%); however, they were group 3 (uncertain significance) mutations (*n* = 98) or mutations not included in the WHO mutational catalogue (*n* = 7). Overall, 88.0% of the isoniazid FN predictions were concordant with TBProfiler. Regarding rifampicin, 62 of the 73 FN predictions made by GenoMycAnalyzer (84.9%) were concordant with those made by TBProfiler.

### Detection of large deletions increases the sensitivity of gDST

**Fig. 3:**
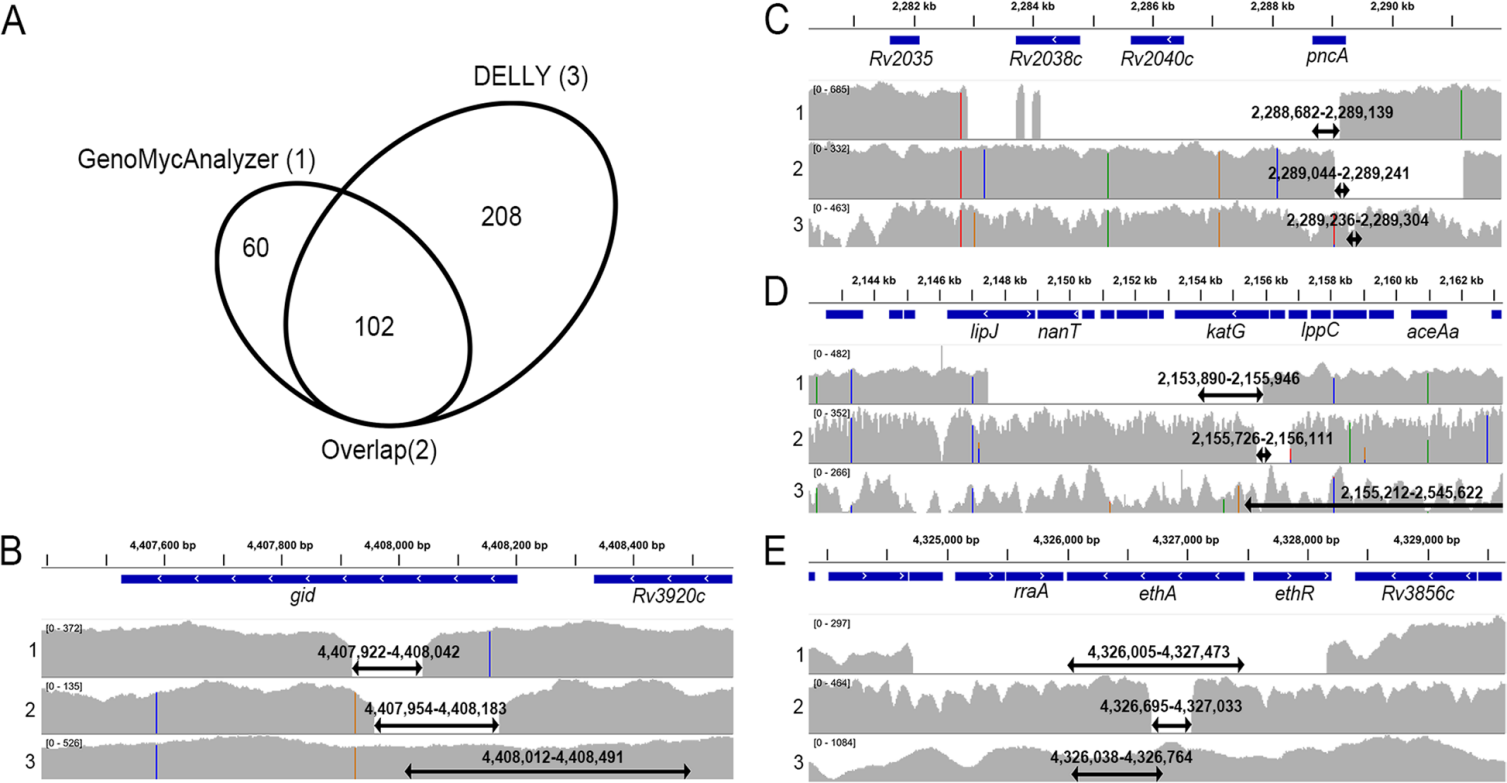
Evaluation of large deletions detected by GenoMycAnalyzer and DELLY. **A** Venn diagram of the large deletions detected by GenoMycAnalyzer and DELLY. Large deletions confirmed as true positive by manual inspection are displayed in gray. **B-D** Examples of large deletions detected in *gid* (**B**), *pncA* (**C**), *katG* (**D**), and *ethA* (**E**). In each example, the top (1), middle (2), and bottom (3) panels represent large deletions detected by GenoMycAnalyzer only, both tools, and DELLY only, respectively. The x-axis represents genomic position, and the y-axis represents sequencing depth. The black arrows indicate the breakpoint of a large deletion. GenoMycAnalyzer detects large deletions at the gene level. Accession numbers for the B-1 to E-3 are SRR958195, ERR718365, SRR6824567, SRR6824340, SRR6824578, ERR040134, SRR6824287, ERR040137, ERR867540, SRR924218, ERR038737, and SRR6824300, respectively

GenoMycAnalyzer identified 25 large deletions in *katG* (0.5%), 58 in *pncA* (1.8%), 69 in *gid* (2.1%), and nine in *ethA* (0.8%). In contrast, when large deletions were predicted using DELLY, the number of large deletions detected was higher than that identified using the GenoMycAnalyzer: 114 in *katG* (2.2%), 36 in *pncA* (1.1%), 90 in *gid* (2.8%), and 33 in *ethA* (2.9%). Most large deletions detected by the GenoMycAnalyzer (63.0%) overlapped with those detected by DELLY, whereas 60 large deletions were specific to the GenoMycAnalyzer (Fig. 3A). Manual inspection using IGV confirmed that all deletions detected by the GenoMycAnalyzer were true positives; however, only 35.1% of the DELLY-specific deletions were true positives (Fig. 3B-D and Additional file 4: Table S7). Comparisons of large deletions with the pDST results revealed that GenoMycAnalyzer had a PPV of 100% for isoniazid, 93.1% for pyrazinamide, 79.7% for streptomycin, and 44.4% for ethionamide, whereas DELLY had a PPV of 71.9% for isoniazid, 83.3% for pyrazinamide, 75.6% for streptomycin, and 24.2% for ethionamide (Additional file 1: Table S8). The sensitivity of the GenoMycAnalyzer gDST, including large deletions, improved to 92.0, 84.5, 83.3, and 80.2% for isoniazid, pyrazinamide, streptomycin, and ethionamide, respectively.

### Species identification performance of GenoMycAnalyzer

To identify MTBC and NTM at the species level, GenoMycAnalyzer was integrated with the Kraken2 and Braken software [26]. To evaluate the accuracy of species identification, we analyzed 1,284 mycobacterial genomes, including 69 isolates of MTBCs and 1,215 isolates of 39 NTM species [20, 37]. The GenoMycAnalyzer reliably distinguished between MTBC and NTM at the species level, achieving an accuracy of 98.8% (1,268 concordant results out of 1,284 isolates), whereas the accuracy of the Kraken2 built-in database was 90.0% (1,156 concordant results out of 1,284 isolates) (Additional file 1: Table S9). Furthermore, the GenoMycAnalyzer successfully identified isolates that had not previously been identified at the species level in the NCBI database. This included an isolate labeled *M. avium complex sp. CF00315-00498*, in which the GenoMycAnalyzer was identified as *M. marseillense*. However, at the subspecies level, the identification performance of GenoMycAnalyzer was significantly lower than its species-level identification performance (508 concordant results out of 803 isolates; 63.3%) (Additional file 1: Table S10). For example, of the 494 isolates reported as *M. abscessus subsp. abscessus*, 296 (59.9%) were identified as the same subspecies, whereas the remaining 198 isolates were identified as *M. abscessus subsp. massiliense* within the same species. Similarly, three out of the 37 isolates reported as *M. intracellulare subsp. chimaera* were identified as *M. intracellulare*. Although incorrect classifications at the subspecies level were identified within the same species (Additional file 1: Table S10), these results suggest that GenoMycAnalyzer subspecies identification may not be optimal. Therefore, GenoMycAnalyzer reports MTBC and NTM at the species level without further subspecies identification.

### Lineage and spoligotype predictions

The lineage of MTBC isolates can be determined based on SNP differences [31, 32]. To evaluate the accuracy of the lineage-prediction module implemented in GenoMycAnalyzer, we analyzed 1,935 MTBC isolates with phylogenetically determined lineages [31]. A total of 84 lineages/sub-lineages were identified, all of which were concordant with previously reported lineages (Additional file 5: Table S11).

GenoMycAnalyzer predicts spoligotypes based on 43 spacer sequences of the direct repeat locus in the MTBC genome. We analyzed 54 MTBC isolates with experimentally determined spoligotypes [38] and identified 20 different spoligotypes. Notably, spoligotype predictions using the GenoMycAnalyzer were concordant with the laboratory-determined spoligotypes for all isolates, resulting in an accuracy of 100% (Additional file 1: Table S12).

## Discussion

In this study, we present a high-confidence automated bioinformatic tool we dubbed the GenoMycAnalyzer. We present extensive validation of this tool, which analyzes MTBC WGS data to provide gDST for 15 antituberculosis drugs using the WHO mutational catalogue [10] and large deletions [33]. GenoMycAnalyzer also offers species-level identification of MTBC and NTM, prediction of the MTBC lineage and spoligotype, and visualization of the detected variants. In a clinical setting, WGS data obtained after patient sample processing could be automatically processed through this pipeline which would present a user-friendly interface and deliver results in standard lab format.

The WHO mutational catalogue is the most comprehensive knowledge base of mutations associated with MTBC drug resistance and assigns a confidence grade to one of five groups for each variant [10]. Group 1 and 2 mutations present in the catalogue accurately predicted resistance and strongly correlated with pDST for most drugs [10], and GenoMycAnalyzer reports strains harboring group 1 or 2 as resistant. One of the limitations of the WHO mutational catalogue is that it has not been validated using an independent dataset [10]. Using the GenoMycAnalyzer, we confirmed the excellent discriminative ability of drug resistance for most antituberculosis drugs, even in an independent large dataset. Specifically, the AUC of GenoMycAnalyzer showed equivalent (rifampicin, isoniazid, ethambutol, moxifloxacin, amikacin, and capreomycin) or better (pyrazinamide, levofloxacin, and kanamycin) prediction performances than those reported by the WHO mutational catalogue, thus confirming the non-inferiority of the GenoMycAnalyzer.

In some cases, it is noteworthy that mutations reported in the WHO mutational catalogue and those detected in this study overlap at the amino acid level but are discordant at the nucleotide level. For example, nucleotide change of *embB* p.Met306Ile conferring ethambutol resistance is ‘c.918G > A’ in the WHO catalogue, whereas ‘c.918G > C’ causing the identical p.Met306Ile was recurrently detected in our ethambutol-resistant isolates. Likewise, *katG* c.944G > C (p.Ser315Thr), *rpsL* c.128A > G (p.Lys43Arg), and *rpoB* c.1349C > T (p.Ser450Leu) identified in this study overlapped at the amino acid level, but were discordant at the nucleotide level. This observation could be related to the inherent redundancy in the genetic code, suggesting that clinicians assessing or diagnosing gDST using the present version of the WHO mutational catalogue should exercise caution to prevent misdiagnoses.

Large deletions lead to the loss of genetic material, can disrupt protein function, and are sporadically observed in drug resistance-related genes such as *katG* and *pncA* [39, 40]. Using WGS data, GenoMycAnalyzer successfully detected large deletions with varying frequencies in *katG* (0.5%), *pncA* (1.8%), *gid* (2.1%), and *ethA* (0.8%). Notably, large deletions were mutually exclusive to resistance-conferring mutations. When large deletions were included, the gDST sensitivity to pyrazinamide and streptomycin increased by 4.9% and 5.4%, respectively. In addition, more than half of the isolates with *pncA* large deletions belonged to the East Asian lineage (lineage 2, 38 of 58 isolates with large *pncA* deletion, 65.5%), consistent with previous findings [28]. These results suggest that the use of WGS data in clinical settings may provide advantages for diagnosing drug-resistant MTBC by simultaneously detecting large deletions and resistance-conferring variants that are difficult to detect using conventional PCR.

Identifying NTMs at the species level is important for effective treatment [41]. Kraken2 is effective in identifying mixed NTM infections, with the highest sensitivity and specificity among the tested analytical tools [42–44]. However, species prediction by Kraken2 using a built-in database revealed that only 90% of the tested isolates matched their known species, and most of the mismatches were due to the absence of NTM sequences within the database (Additional file 1: Table S9). These include *M. colombiense*, *M. asiaticum*, and *M. malmoense*, which cause human infections [45–47]. In contrast, GenoMycAnalyzer uses a custom-built database that adds 107 NTM sequences, and its species identification accuracy is as high as 98.8%. The GenoMycAnalyzer has two additional functionalities that are important for mycobacterial analysis. First, the GenoMycAnalyzer includes a molecular typing function, and large-scale validation confirmed accurate lineage and spoligotype predictions. These features may be useful for epidemiological surveillance of mycobacteria [31, 48]. Second, the GenoMycAnalyzer integrates the IGV browser to visualize the detected variants. Manual inspection using IGV may allow investigators to assess variants efficiently, serving as a potent method for validating variant calls and reducing false positives while confirming true findings [15].

An inherent limitation of this study is that the gDST predictions between software programs may be inconsistent. These discrepancies could arise from the cohort used for the analysis, incorrect pDST data, lack of standardization of the bioinformatics pipeline, or different knowledge databases. Given that most of the differences between GenoMycAnalyzer and TBProfiler results were due to group 3 variants, further evaluation of group 3 mutations is required. In addition, we did not evaluate the four drugs because of a lack of resistance-conferring mutations or phenotypically resistant isolates. Further studies in a larger cohort are required to discover novel resistance-conferring mutations and elucidate the underlying resistance mechanisms. GenoMycAnalyzer includes a function for users to edit the knowledge database, which serves as a valuable bridge until regular database updates occur. Finally, although the GenoMycAnalyzer accurately distinguished clinically important mycobacteria at the species level, further efforts are needed to improve the accuracy of subspecies identification.

## Conclusions

GenoMycAnalyzer software provides flexible and rapid analysis of WGS data from the Illumina platform to predict species, drug resistance, and molecular profiles with high accuracy. GenoMycAnalyzer also offers both web-based and GUI interfaces, which can help biologists with limited access to high-performance computing systems or limited bioinformatics skills. Ultimately, by streamlining the interpretation of WGS data, the GenoMycAnalyzer has the potential to significantly impact TB management and contribute to global efforts to combat this infectious disease.

## Availability and requirements

Project name: GenoMycAnalyzer.

Project home page: https://www.mycochase.org/

Operating system(s): Windows, Linux, MacOS.

Programming language: Python, SQL, JavaScript, HTML.

Other requirements: None.

License: GNU General Public License.

Any restrictions to use by non-academics: None.

### Supplementary Information

**Additional file 1:** **Table S1**. Major functions of GenoMycAnalyzer compared to other whole genome sequencing analysis tools. **Table S2**. NTM genomes newly added to custom-built database. **Table S3**. Drugs and resistance-associated genes investigated in this study. **Table S5**. Significance of difference in AUC between two datasets. **Table S8**. Comparison of large deletion and pDST results. **Table S9**. GenoMycAnalyzer species predictions compared to those of the NCBI report. **Table S10**. GenoMycAnalyzer sub-species predictions compared to those of the NCBI report. **Table S12**. Comparison of GenoMycAnalyzer spoligotype predictions with reported spoligotype for 54 MTBC isolates.

**Additional file 2:** **Table S4**. Sample accession identifier and corresponding metadata.

**Additional file 3:** **Table S6**. Discordant in resistance predictions.

**Additional file 4:** **Table S7**. Large deletions detected in four target genes.

**Additional file 5:** **Table S11**. Comparison of GenoMycAnalyser's lineage predictions with reported lineages for 1,935 MTBC isolates.

## Data Availability

To evaluate the performance of the GenoMycAnalyzer, we collected a dataset of 8,259 Illumina raw sequence reads for which laboratory-based metadata were available from public datasets. Raw sequence data were downloaded from the European Nucleotide Archive (ENA, https://www.ebi.ac.uk/ena/browser/home), the Sequence Read Archive (SRA, https://www.ncbi.nlm.nih.gov/sra), or the DDBJ Sequence Read Archive (https://www.ddbj.nig.ac.jp/dra/index-e.html). Accession numbers are listed in Additional file 2: Table S3. The source code for the data processing pipeline is available in the GitHub repository at https://github.com/IRCGP-Lab/GenoMycAnalyzer_Source. The GenoMycAnalyzer is accessible at https://www.mycochase.org/.

## Abbreviations

TB: Tuberculosis
WGS: Whole-genome sequencing
MTBC: *M**ycobacterium** tuberculosis* Complex
NTM: Non-tuberculous mycobacteria
gDST: Genotypic drug susceptibility testing
pDST: Phenotypic drug susceptibility testing
MAC: *Mycobacterium avium* Complex
RAV: Resistance-associated variants
WHO: World Health Organization
VAF: Variant allele frequency
IGV: Integrative Genomics Viewer
SNP: Single-nucleotide polymorphism
PPV: Positive predictive value
NPV: Negative predictive value
ROC: Receiver operating characteristic
AUC: Area under the curve
ENA: European Nucleotide Archive
SRA: Sequence Read Archive
MDR-TB: Multidrug-resistant tuberculosis
XDR-TB: Extensively drug-resistant tuberculosis
PCR: Polymerase chain reaction

## References

[1] Tortoli E, Meehan CJ, Grottola A, Fregni Serpini G, Fabio A, Trovato A (2019). Genome-based taxonomic revision detects a number of synonymous taxa in the genus Mycobacterium. Infect Genet Evol.

[2] Bagcchi S (2023). WHO's Global Tuberculosis Report 2022. Lancet Microbe.

[3] Mukherjee JS, Rich ML, Socci AR, Joseph JK, Viru FA, Shin SS (2004). Programmes and principles in treatment of multidrug-resistant tuberculosis. Lancet.

[4] Stout JE, Koh WJ, Yew WW (2016). Update on pulmonary disease due to non-tuberculous mycobacteria. Int J Infect Dis.

[5] Ratnatunga CN, Lutzky VP, Kupz A, Doolan DL, Reid DW, Field M (2020). The Rise of Non-Tuberculosis Mycobacterial Lung Disease. Front Immunol.

[6] Cowman S, van Ingen J, Griffith DE, Loebinger MR (2019). Non-tuberculous mycobacterial pulmonary disease. Eur Respir J..

[7] Nguyen TNA, Anton-Le Berre V, Banuls AL, Nguyen TVA (2019). Molecular Diagnosis of Drug-Resistant Tuberculosis. Front Microbiol.

[8] He Y, Gong Z, Zhao X, Zhang D, Zhang Z (2020). Comprehensive Determination of Mycobacterium tuberculosis and Nontuberculous Mycobacteria From Targeted Capture Sequencing. Front Cell Infect Microbiol.

[9] Dookie N, Khan A, Padayatchi N, Naidoo K (2022). Application of Next Generation Sequencing for Diagnosis and Clinical Management of Drug-Resistant Tuberculosis: Updates on Recent Developments in the Field. Front Microbiol.

[10] Walker TM, Miotto P, Koser CU, Fowler PW, Knaggs J, Iqbal Z (2022). The 2021 WHO catalogue of Mycobacterium tuberculosis complex mutations associated with drug resistance: A genotypic analysis. Lancet Microbe.

[11] Beckert P, Hillemann D, Kohl TA, Kalinowski J, Richter E, Niemann S, Feuerriegel S (2012). rplC T460C identified as a dominant mutation in linezolid-resistant Mycobacterium tuberculosis strains. Antimicrob Agents Chemother.

[12] Nimmo C, Millard J, van Dorp L, Brien K, Moodley S, Wolf A (2020). Population-level emergence of bedaquiline and clofazimine resistance-associated variants among patients with drug-resistant tuberculosis in southern Africa: a phenotypic and phylogenetic analysis. Lancet Microbe.

[13] Reichmuth ML, Homke R, Zurcher K, Sander P, Avihingsanon A, Collantes J (2020). Natural Polymorphisms in Mycobacterium tuberculosis Conferring Resistance to Delamanid in Drug-Naive Patients. Antimicrob Agents Chemother..

[14] Rossen JWA, Friedrich AW, Moran-Gilad J, Genomic ESGf, Molecular D (2018). Practical issues in implementing whole-genome-sequencing in routine diagnostic microbiology. Clin Microbiol Infect.

[15] Robinson JT, Thorvaldsdottir H, Winckler W, Guttman M, Lander ES, Getz G, Mesirov JP (2011). Integrative genomics viewer. Nat Biotechnol.

[16] Steiner A, Stucki D, Coscolla M, Borrell S, Gagneux S (2014). KvarQ: targeted and direct variant calling from fastq reads of bacterial genomes. BMC Genomics.

[17] Feuerriegel S, Schleusener V, Beckert P, Kohl TA, Miotto P, Cirillo DM (2015). PhyResSE: a Web Tool Delineating Mycobacterium tuberculosis Antibiotic Resistance and Lineage from Whole-Genome Sequencing Data. J Clin Microbiol.

[18] Hunt M, Bradley P, Lapierre SG, Heys S, Thomsit M, Hall MB (2019). Antibiotic resistance prediction for Mycobacterium tuberculosis from genome sequence data with Mykrobe. Wellcome Open Res.

[19] Phelan JE, O'Sullivan DM, Machado D, Ramos J, Oppong YEA, Campino S (2019). Integrating informatics tools and portable sequencing technology for rapid detection of resistance to anti-tuberculous drugs. Genome Med.

[20] Yang T, Gan M, Liu Q, Liang W, Tang Q, Luo G (2022). SAM-TB: a whole genome sequencing data analysis website for detection of Mycobacterium tuberculosis drug resistance and transmission. Brief Bioinform..

[21] Ezewudo M, Borens A, Chiner-Oms A, Miotto P, Chindelevitch L, Starks AM (2018). Integrating standardized whole genome sequence analysis with a global Mycobacterium tuberculosis antibiotic resistance knowledgebase. Sci Rep.

[22] Martin M (2011). Cutadapt removes adapter sequences from high-throughput sequencing reads. EMBnet journal.

[23] Andrews S (2010). FastQC: a quality control tool for high throughput sequence data.

[24] Li H, Durbin R (2009). Fast and accurate short read alignment with Burrows-Wheeler transform. Bioinformatics.

[25] Li H, Handsaker B, Wysoker A, Fennell T, Ruan J, Homer N (2009). The Sequence Alignment/Map format and SAMtools. Bioinformatics.

[26] Lu J, Rincon N, Wood DE, Breitwieser FP, Pockrandt C, Langmead B (2022). Metagenome analysis using the Kraken software suite. Nat Protoc.

[27] Xia E, Teo YY, Ong RT (2016). SpoTyping: fast and accurate in silico Mycobacterium spoligotyping from sequence reads. Genome Med.

[28] Kim NY, Kim DY, Chu J, Jung SH (2023). pncA Large Deletion is the Characteristic of Pyrazinamide-Resistant Mycobacterium tuberculosis belonging to the East Asian Lineage. Infect Chemother.

[29] Danecek P, Bonfield JK, Liddle J, Marshall J, Ohan V, Pollard MO (2021). Twelve years of SAMtools and BCFtools. Gigascience..

[30] Cingolani P, Platts A, le Wang L, Coon M, Nguyen T, Wang L (2012). A program for annotating and predicting the effects of single nucleotide polymorphisms, SnpEff: SNPs in the genome of Drosophila melanogaster strain w1118; iso-2; iso-3. Fly (Austin).

[31] Napier G, Campino S, Merid Y, Abebe M, Woldeamanuel Y, Aseffa A (2020). Robust barcoding and identification of Mycobacterium tuberculosis lineages for epidemiological and clinical studies. Genome Med.

[32] Coll F, McNerney R, Guerra-Assuncao JA, Glynn JR, Perdigao J, Viveiros M (2014). A robust SNP barcode for typing Mycobacterium tuberculosis complex strains. Nat Commun.

[33] Gomes LC, Campino S, Marinho CRF, Clark TG, Phelan JE (2021). Whole genome sequencing reveals large deletions and other loss of function mutations in Mycobacterium tuberculosis drug resistance genes. Microb Genom..

[34] Mahmoud M, Gobet N, Cruz-Davalos DI, Mounier N, Dessimoz C, Sedlazeck FJ (2019). Structural variant calling: the long and the short of it. Genome Biol.

[35] Rausch T, Zichner T, Schlattl A, Stutz AM, Benes V, Korbel JO (2012). DELLY: structural variant discovery by integrated paired-end and split-read analysis. Bioinformatics.

[36] Krzywinski M, Schein J, Birol I, Connors J, Gascoyne R, Horsman D (2009). Circos: an information aesthetic for comparative genomics. Genome Res.

[37] Clark TG, Mallard K, Coll F, Preston M, Assefa S, Harris D (2013). Elucidating emergence and transmission of multidrug-resistant tuberculosis in treatment experienced patients by whole genome sequencing. PLoS ONE.

[38] Hijikata M, Keicho N, Duc LV, Maeda S, Hang NTL, Matsushita I, Kato S (2017). Spoligotyping and whole-genome sequencing analysis of lineage 1 strains of Mycobacterium tuberculosis in Da Nang. Vietnam PLoS One.

[39] Suzuki Y, Suzuki A, Tamaru A, Katsukawa C, Oda H (2002). Rapid detection of pyrazinamide-resistant Mycobacterium tuberculosis by a PCR-based in vitro system. J Clin Microbiol.

[40] Ramaswamy SV, Reich R, Dou SJ, Jasperse L, Pan X, Wanger A (2003). Single nucleotide polymorphisms in genes associated with isoniazid resistance in Mycobacterium tuberculosis. Antimicrob Agents Chemother.

[41] Daley CL, Glassroth J (2014). Treatment of pulmonary nontuberculous mycobacterial infections: many questions remain. Ann Am Thorac Soc.

[42] Yoon JK, Kim TS, Kim JI, Yim JJ (2020). Whole genome sequencing of Nontuberculous Mycobacterium (NTM) isolates from sputum specimens of co-habiting patients with NTM pulmonary disease and NTM isolates from their environment. BMC Genomics.

[43] Khieu V, Ananta P, Kaewprasert O, Laohaviroj M, Namwat W, Faksri K (2021). Whole-Genome Sequencing Analysis to Identify Infection with Multiple Species of Nontuberculous Mycobacteria. Pathogens..

[44] Davidovich N, Makhon A, Zizelski Valenci G, Dveyrin Z, Yahav T, Pretto T (2023). Identification of Mycobacterium pseudoshottsii in the Eastern Mediterranean. Microbiol Spectr.

[45] Hoefsloot W, van Ingen J, de Lange WC, Dekhuijzen PN, Boeree MJ, van Soolingen D (2009). Clinical relevance of Mycobacterium malmoense isolation in The Netherlands. Eur Respir J.

[46] Grech M, Carter R, Thomson R (2010). Clinical significance of Mycobacterium asiaticum isolates in Queensland. Australia J Clin Microbiol.

[47] Yu X, Jiang W (2021). Mycobacterium colombiense and Mycobacterium avium Complex Causing Severe Pneumonia in a Patient with HIV Identified by a Novel Molecular-Based Method. Infect Drug Resist.

[48] Genestet C, Hodille E, Bernard A, Vallee M, Lina G, Le Meur A (2022). Consistency of Mycobacterium tuberculosis Complex Spoligotyping between the Membrane-Based Method and In Silico Approach. Microbiol Spectr.

