# Supplementary material for: GenoMycAnalyzer: a web-based tool for species and drug resistance prediction for Mycobacterium genomes
Source: BMC Genomics. 2024 Apr 20;25:387. doi: 10.1186/s12864-024-10320-3 (PMC11031912; doi:10.1186/s12864-024-10320-3)
Supplement: Supplementary file 1 — Additional file 1: Table S1. Major functions of GenoMycAnalyzer compared to other whole genome sequencing analysis tools. Table S2. NTM genomes newly added to custom-built database. Table S3. Drugs and resistance-associated genes investigated in this study. Table S5. Significance of difference in AUC between two datasets. Table S8. Comparison of large deletion and pDST results. Table S9. GenoMycAnalyzer species predictions compared to those of the NCBI report. Table S10. GenoMycAnalyzer sub-species predictions compared to those of the NCBI report. Table S12. Comparison of GenoMycAnalyzer spoligotype predictions with reported spoligotype for 54 MTBC isolates. [file 12864_2024_10320_MOESM1_ESM.pdf]

Supplementary Tables

Table S1. Major functions of GenoMycAnalyzer compared to other whole genome sequencing analysis tools.

Table S2. NTM genomes newly added to custom-built database.

Table S3. Drugs and resistance-associated genes investigated in this study.

Table S5. Significance of difference in AUC between two datasets.

Table S8. Comparison of large deletion and pDST results.

Table S9. GenoMycAnalyzer species predictions compared to those of the NCBI report.

Table S10. GenoMycAnalyzer sub-species predictions compared to those of the NCBI report.

Table S12. Comparison of GenoMycAnalyzer spoligotype predictions with reported spoligotype for 54 MTBC isolates.

Table S1. Major functions of GenoMycAnalyzer compared to other whole genome sequencing analysis tools.

Function	GenoMycAnalyzer	KvarQ	PhyResSE	Mykrobe	ReSeqTB-UVP	TBProfiler	SAM-TB
MTBC/NTM identification	Yes	No (Only TB)	No (Only TB)	Yes	No (Only TB)	No (Only TB)	Yes
Genotypic DST*	INH, RIF, EMB, PZA, STM, KAN, AMK, CAP, MFX, LFX, ETO, LZD, CFZ, BDQ, DLM	INH, RIF, EMB, STM, KAN, AMK, FQs	INH, RIF, EMB, PZA, STM, KAN, AMK, CAP, FQs, ETO, PAS, LZD	INH, RIF, EMB, STM, KAN, AMK, CAP, MFX, OFX	INH, RIF, EMB, PZA, STM, KAN, AMK, CAP, FQs	INH, RIF, EMB, PZA, STM, KAN, AMK, CAP, FQs, ETO, PAS, LZD, CFZ, BDQ, DLM	INH, RIF, EMB, PZA, STM, KAN, AMK, CAP, MFX, OFX, ETO, PAS, LZD, CFZ, BDQ, DLM
Lineage prediction	Yes	Yes	Yes	Yes	Yes	Yes	Yes
Spoligotype prediction	Yes	No	No	No	No	Yes	No
Large deletion prediction	Yes	No	No	No	Yes	No	No
Variant visualization (IGV)	Yes	No	No	No	No	Yes	No
Web-based	Yes	No	Yes (no longer available)	No	No	Yes	Yes

* DST, drug susceptibility testing; AMK, amikacin; BDQ, bedaquiline; CAP, capreomycin; CFZ, clofazimine; DLM, delamanid; EMB, ethambutol; ETO, ethionamide; FQs, fluoroquinolones; INH, isoniazid; KAN, kanamycin; LFX, levofloxacin; LZD, linezolid; MFX, moxifloxacin; OFX, ofloxacin; PTO, prothionamide; PZA, pyrazinamide; RIF, rifampicin; STM, streptomycin

Table S2. NTM genomes newly added to custom-built database

Species name	NCBI RefSeq assembly
Mycobacterium alsense	GCF_001666815.1_ASM166681v1
Mycobacterium alsense	GCF_001672935.1_ASM167293v1
Mycobacterium alsense	GCF_002086635.1_ASM208663v1
Mycobacterium alsense	GCF_025823205.1_ASM2582320v1
Mycobacterium asiaticum	GCF_000613245.1_PRJEB5743_assembly_1
Mycobacterium asiaticum	GCF_001668675.1_ASM166867v1
Mycobacterium asiaticum	GCF_001669345.1_ASM166934v1
Mycobacterium asiaticum	GCF_001672925.1_ASM167292v1
Mycobacterium asiaticum	GCF_001673165.1_ASM167316v1
Mycobacterium asiaticum	GCF_001673315.1_ASM167331v1
Mycobacterium asiaticum	GCF_001673345.1_ASM167334v1
Mycobacterium asiaticum	GCF_001673365.1_ASM167336v1
Mycobacterium asiaticum	GCF_002086545.1_ASM208654v1
Mycobacterium asiaticum	GCF_001673635.1_ASM167363v1
Mycobacterium kyorinense	GCF_000759695.1_ASM75969v1
Mycobacterium kyorinense	GCF_001439515.1_ASM143951v1
Mycobacterium kyorinense	GCF_001672775.1_ASM167277v1
Mycobacterium kyorinense	GCF_002101735.1_ASM210173v1
Mycobacterium heraklionense	GCF_001021505.1_ASM102150v1
Mycobacterium heraklionense	GCF_019645815.1_ASM1964581v1
Mycobacterium colombiense	GCF_000222105.3_ASM22210v4
Mycobacterium colombiense	GCF_001665835.1_ASM166583v1
Mycobacterium colombiense	GCF_001667905.1_ASM166790v1
Mycobacterium colombiense	GCF_001672755.1_ASM167275v1
Mycobacterium colombiense	GCF_001673005.1_ASM167300v1
Mycobacterium colombiense	GCF_001673015.1_ASM167301v1
Mycobacterium colombiense	GCF_001673075.1_ASM167307v1
Mycobacterium colombiense	GCF_001673085.1_ASM167308v1
Mycobacterium colombiense	GCF_001673175.1_ASM167317v1
Mycobacterium colombiense	GCF_001673195.1_ASM167319v1
Mycobacterium colombiense	GCF_001673505.1_ASM167350v1
Mycobacterium colombiense	GCF_001953985.1_ASM195398v1
Mycobacterium colombiense	GCF_001954055.1_ASM195405v1
Mycobacterium colombiense	GCF_001954075.1_ASM195407v1
Mycobacterium colombiense	GCF_001954115.1_ASM195411v1
Mycobacterium colombiense	GCF_002105755.1_ASM210575v1
Mycobacterium colombiense	GCF_003284935.1_ASM328493v1
Mycobacterium colombiense	GCF_003284975.1_ASM328497v1
Mycobacterium colombiense	GCF_023218095.1_ASM2321809v1
Mycobacterium colombiense	GCF_900161855.1_PRJEB19244
Mycobacterium lehmannii	GCF_001499925.1_ASM149992v1
Mycobacterium lehmannii	GCF_002245535.1_ASM224553v1
Mycobacterium malmoense	GCF_001686665.1_ASM168666v1
Mycobacterium malmoense	GCF_001686725.1_ASM168672v1
Mycobacterium malmoense	GCF_001686735.1_ASM168673v1
Mycobacterium malmoense	GCF_001686745.1_ASM168674v1
Mycobacterium malmoense	GCF_001686825.1_ASM168682v1
Mycobacterium malmoense	GCF_001870845.1_ASM187084v1
Mycobacterium malmoense	GCF_002086305.1_ASM208630v1
Mycobacterium malmoense	GCF_019645855.1_ASM1964585v1
Mycobacterium malmoense	GCF_022558505.1_ASM2255850v1
Mycobacterium_porcinum	GCF_025823105.1_ ASM2582310v1
Mycobacterium scrofulaceum	GCF_001667885.1_ASM166788v1
Mycobacterium scrofulaceum	GCF_001672575.1_ASM167257v1
Mycobacterium scrofulaceum	GCF_002086735.1_ASM208673v1

Mycobacterium szulgai	GCF_002116635.1_ASM211663v1
Mycobacterium szulgai	GCF_025821385.1_ASM2582138v1
Mycobacterium vulneris	GCF_002104765.1_ASM210476v1
Mycolicibacter arupensis	GCF_000970885.2_ASM97088v2
Mycolicibacter arupensis	GCF_002086515.1_ASM208651v1
Mycolicibacter arupensis	GCF_008373105.1_ASM837310v1
Mycolicibacter arupensis	GCF_025822685.1_ASM2582268v1
Mycolicibacterium conceptionense	GCF_000233935.1_Mfort_1.0
Mycolicibacterium conceptionense	GCF_001077745.1_ASM107774v1
Mycolicibacterium conceptionense	GCF_001665335.1_ASM166533v1
Mycolicibacterium conceptionense	GCF_001667225.1_ASM166722v1
Mycolicibacterium conceptionense	GCF_001667235.1_ASM166723v1
Mycolicibacterium conceptionense	GCF_001667305.1_ASM166730v1
Mycolicibacterium conceptionense	GCF_001667965.1_ASM166796v1
Mycolicibacterium conceptionense	GCF_001673265.1_ASM167326v1
Mycolicibacterium conceptionense	GCF_001954125.1_ASM195412v1
Mycolicibacterium conceptionense	GCF_001954205.1_ASM195420v1
Mycolicibacterium conceptionense	GCF_001954235.1_ASM195423v1
Mycolicibacterium conceptionense	GCF_002102065.1_ASM210206v1
Mycolicibacterium conceptionense	GCF_025823065.1_ASM2582306v1
Mycolicibacterium elephantis	GCF_001005175.1_ASM100517v1
Mycolicibacterium elephantis	GCF_001665365.1_ASM166536v1
Mycolicibacterium elephantis	GCF_001665755.1_ASM166575v1
Mycolicibacterium elephantis	GCF_001667535.1_ASM166753v1
Mycolicibacterium elephantis	GCF_002086605.1_ASM208660v1
Mycolicibacterium elephantis	GCF_004014805.1_ASM401480v1
Mycolicibacterium elephantis	GCF_025822495.1_ASM2582249v1
Mycolicibacterium novocastrense	GCF_001499825.1_ASM149982v1
Mycolicibacterium novocastrense	GCF_001499835.1_ASM149983v1
Mycolicibacterium novocastrense	GCF_001499845.1_ASM149984v1
Mycolicibacterium novocastrense	GCF_001570485.1_ASM157048v1
Mycolicibacterium novocastrense	GCF_025822165.1_ASM2582216v1
Mycolicibacterium peregrinum	GCF_001665625.1_ASM166562v1
Mycolicibacterium peregrinum	GCF_001403655.1_GCF_001403655.1
Mycolicibacterium peregrinum	GCF_001665785.1_ASM166578v1
Mycolicibacterium peregrinum	GCF_001667205.1_ASM166720v1
Mycolicibacterium peregrinum	GCF_002102345.1_ASM210234v1
Mycolicibacterium peregrinum	GCF_002198105.1_ASM219810v1
Mycolicibacterium peregrinum	GCF_004721025.1_ASM472102v1
Mycolicibacterium peregrinum	GCF_004721035.1_ASM472103v1
Mycolicibacterium peregrinum	GCF_025822425.1_ASM2582242v1
Mycolicibacterium setense	GCF_000805375.1_ASM80537v1
Mycolicibacterium setense	GCF_000805385.1_ASM80538v1
Mycolicibacterium setense	GCF_001665445.1_ASM166544v1
Mycolicibacterium setense	GCF_900236745.1_PRJEB23414
Mycolicibacterium setense	GCF_025821545.1_ASM2582154v1
Mycolicibacter sinensis	GCF_000214155.1_ASM21415v1
Mycolicibacter sinensis	GCF_001667375.1_ASM166737v1
Mycolicibacter sinensis	GCF_001667395.1_ASM166739v1
Mycolicibacter sinensis	GCF_001667945.1_ASM166794v1
Mycolicibacter sinensis	GCF_001672735.1_ASM167273v1
Mycolicibacter sinensis	GCF_001673565.1_ASM167356v1

Table S3. Drugs and resistance-associated genes investigated in this study.

Antituberculosis drug	Resistance-associated genes*
Isoniazid	*fabG1, katG, Rv0485, mshA, Rb11258c, udgB, Rv1482c, inhA, ndh, furA, ahpC, Rv2751, rnj, dapA*
Rifampicin	*rpoB, Rv2751, rnj, dapA, rpoC, rpoA, rpsD*
Ethambutol	*embA, embB, embR, Rv3788, Rv3789, dprE1, aftA, embC, ubiA, glf, pirG*
Pyrazinamide	*pncA, Rv1258c, udgB, PPE35, Rv3236c, Rv3237c, clpC1, panD, panC, Rv3603c*
Levofloxacin	*gyrA, gyrB, Rv0007*
Moxifloxacin	*gyrA, gyrB, Rv0007*
Linezolid	*rplC, rpsJ, murA, mcr3, rrs, rrl*
Bedaquiline	*mmpL5, mmpS5, Rv0678, atpB, atpE, Rv1978, Rv1979c, mpt64, pepQ*
Clofazimine	*mmpL5, mmpS5, Rv0678, Rv1979c, mpt64, pepQ, Rv2536*
Delamanid	*ddn, fgd1, fbiA, fbiB, fbiC, fbiD, fadA5*
Amikacin	*rrs, eis, murA, mcr3, Rv0528, ccsA, prfB, fprA, whiB7, aftB, ubiA, Rv3861, whiB6, Rv3863*
Capreomycin	*rrs, tlyA, murA, mcr3, Rv0528, ccsA, prfB, fprA, aftB, ubiA, Rv3861, whiB6, Rv3863, Rv1692, Rv1693*
Kanamycin	*rrs, eis, murA, mcr3, whiB7*
Streptomycin	*rrs, rpsL, gid, Rv1258c, murA, mcr3, whiB7, Rv3861, whiB6, Rv3863, udgB, Rv0681*
Ethionamide	*fabG1, ethA, inhA, mshA, ndh, mymA, Rv0485, ethR, Rv1482c*

*Resistance-associated genes include all of groups 1-5 of the WHO mutational catalogue.

Table S5. Significance of difference in AUC between two datasets.

Drug	WHO mutational catalogue dataset			GenoMycAnalyzer dataset			Comparison			
	Area	95% confidence interval		Area	95% confidence interval		AUC difference	p-value	95% confidence interval	
		Lower bound	Upper bound		Lower bound	Upper bound			Lower bound	Upper bound
Rifampicin	0.960	0.949	0.963	0.964	0.957	0.972	0.004	0.240	-0.003	0.011
Isoniazid	0.948	0.944	0.961	0.944	0.931	0.948	-0.004	0.317	-0.013	0.004
Ethambutol	0.900	0.878	0.904	0.909	0.896	0.922	0.009	0.179	-0.004	0.022
Pyrazinamide	0.856	0.805	0.843	0.887	0.900	0.938	0.032	0.001	0.013	0.051
Levofloxacin	0.913	0.817	0.894	0.971	0.932	1.009	0.057	0.004	0.019	0.096
Moxifloxacin	0.896	0.89	0.955	0.870	0.836	0.903	-0.027	0.105	-0.059	0.006
Amikacin	0.882	0.827	0.888	0.906	0.872	0.955	0.025	0.121	-0.006	0.055
Capreomycin	0.838	0.784	0.857	0.856	0.837	0.900	0.018	0.338	-0.019	0.054
Kanamycin	0.858	0.746	0.785	0.950	1.023	1.062	0.093	<0.001	0.073	0.112
Streptomycin	0.889	0.895	0.930	0.866	0.825	0.860	-0.023	0.009	-0.041	-0.006
Ethionamide	0.836	0.847	0.909	0.794	0.759	0.829	-0.042	0.008	-0.073	-0.011

Table S8. Comparison of large deletion and pDST results.

Drug	Target gene	GenoMycAnalyzer		Delly	
		Large deletion (%)	Positive predictive value	Large deletion (%)	Positive predictive value
Isoniazid	*katG*	24 (0.5%)	100.0%	114 (2.2%)	71.9%
Pyrazinamide	*pncA*	58 (1.8%)	93.1%	36 (1.1%)	83.3%
Streptomycin	*gid*	69 (2.1%)	79.7%	90 (2.8%)	75.6%
Ethionamide	*ethA*	9 (0.8%)	44.4%	33 (2.9%)	24.2%

Table S9. GenoMycAnalyzer species predictions compared to those of the NCBI report.

Complex	Species	# of isolates	GenoMycAnalyzer			Kraken2		
			Concordant identification	Discordant identification (%)		Concordant identification	Discordant identification (%)	
				In same complex	Not in same complex		In same complex	Not in same complex
M. tuberculosis complex	*M. tuberculosis*	69	69 (100%)	0	0	69 (100%)	0	0
M. abscessus complex	*M. abscessus*	634	634 (100%)	0	0	634 (100%)	0	0
M. avium complex	*M. avium*	212	211 (99.5%)	1	0	211 (99.5%)	1	0
M. avium complex	*M. colombiense*	37	37 (100%)	0	0	0(0%)	0	37
M. avium complex	*M. intracellulare*	160	160 (100%)	0	0	160 (100%)	0	0
M. avium complex	*M. mantenii*	10	10 (100%)	0	0	10 (100%)	0	0
M. avium complex	*M. marseillense*	4	4 (100%)	0	0	4 (100%)	0	0
-	*M. fortuitum*	38	38 (100%)	0	0	38 (100%)	0	0
-	*M. gordonae*	18	11 (61%)	0	7	11 (56%)	0	7
-	*M. kansasii*	3	2 (67%)	0	1	2 (67%)	0	1
-	*M. malmoense*	12	12 (100%)	0	0	2 (17%)	0	10
-	*M. marinum*	2	2 (100%)	0	0	2 (100%)	0	0
-	*M. xenopi*	2	2 (100%)	0	0	2 (100%)	0	0
-	*M. alsense*	6	6 (100%)	0	0	0 (0%)	0	6
-	*M. asiaticum*	18	18 (100%)	0	0	0 (0%)	0	18
-	*M. kubicae*	1	1 (100%)	0	0	1 (100%)	0	0
-	*M. kyorinense*	2	2 (100%)	0	0	0 (0%)	0	2
-	*M. lehmannii*	1	1 (100%)	0	0	0 (0%)	0	1
-	*M. lentiflavum*	1	1 (100%)	0	0	1 (100%)	0	0
-	*M. porcinum*	3	3 (100%)	0	0	0 (0%)	0	3
-	*M. scrofulaceum*	4	4 (100%)	0	0	0 (0%)	0	4
-	*M. szulgai*	7	2 (29%)	0	5	0 (0%)	0	7
-	*M. chelonae*	4	4 (100%)	0	0	4 (100%)	0	0
-	*M. sinensis*	8	8 (100%)	0	0	0 (0%)	0	8
-	*M. celeriflavum*	1	1 (100%)	0	0	1 (100%)	0	0
-	*M. conceptionense*	10	10 (100%)	0	0	0 (0%)	0	10
-	*M. elephantis*	4	4 (100%)	0	0	0 (0%)	0	4
-	*M. monacense*	2	2 (100%)	0	0	2 (100%)	0	0
-	*M. mucogenicum*	4	2 (50%)	0	2	2 (50%)	0	2
-	*M. novocastrense*	3	3 (100%)	0	0	0 (0%)	0	3
-	*M. peregrinum*	3	3 (100%)	0	0	0 (0%)	0	3
-	*M. setense*	1	1 (100%)	0	0	0 (0%)	0	1

Table S10. GenoMycAnalyzer sub-species predictions compared to those of the NCBI report.

Sub-species	# of isolates	GenoMycAnalyzer			Kraken2		
		Concordant identification	Discordant identification (%)		Concordant identification	Discordant identification (%)	
			In same species	Not in same species		In same species	Not in same species
M. abscessus subsp. abscessus	494	296 (60%)	198	0	262 (53%)	236	0
M. abscessus subsp. bolletii	11	4 (36%)	7	0	3 (27%)	8	0
M. abscessus subsp. massiliense	114	100 (88%)	14	0	103 (90%)	11	0
M. avium subsp. hominissuis	80	68 (85%)	12	0	70 (88%)	10	0
M. intracellulare subsp. chimaera	37	34 (92%)	3	0	34 (91%)	3	0
M. intracellulare subsp. intracellulare	61	4 (7%)	57	0	28 (45%)	33	0
M. intracellulare subsp. yongonense	6	2 (33%)	4	0	2 (33%)	4	0

Table S12. Comparison of GenoMycAnalyzer spoligotype predictions with reported spoligotype for 54 MTBC isolates.

Sample Accession	Reported spoligotype	Predicted spoligotype	Concordance
DRR099683	777777774413771	777777774413771	Yes
DRR099684	760000074413771	760000074413771	Yes
DRR099685	777777774413771	777777774413771	Yes
DRR099686	777777000000011	777777000000011	Yes
DRR099687	777737774413771	777737774413771	Yes
DRR099688	775777774413771	775777774413771	Yes
DRR099689	777777774413011	777777774413011	Yes
DRR099690	777737774413731	777737774413731	Yes
DRR099691	777737774413771	777737774413771	Yes
DRR099692	777777777413771	777777777413771	Yes
DRR099693	777777774413771	777777774413771	Yes
DRR099694	777777000000011	777777000000011	Yes
ERR036201	700777747413771	700777747413771	Yes
ERR036213	700777747413771	700777747413771	Yes
ERR036217	700777747413771	700777747413771	Yes
ERR036222	700777747413771	700777747413771	Yes
ERR036223	757777777413731	757777777413731	Yes
ERR036230	700777746013771	700777746013771	Yes
ERR036236	700777747413771	700777747413771	Yes
ERR037476	700777747413771	700777747413771	Yes
ERR037491	477777777413071	477777777413071	Yes
ERR037511	700777747413771	700777747413771	Yes
ERR234207	777777000000011	777777000000011	Yes
ERR234238	777777774413771	777777774413771	Yes
ERR234240	777777774413771	777777774413771	Yes
ERR234241	777777774413771	777777774413771	Yes
ERR245662	757777777413731	757777777413731	Yes
ERR688020	777377700003531	777377700003531	Yes
ERR688024	477777777413071	477777777413071	Yes
ERR688038	400000037413071	400000037413071	Yes
ERR688039	477777777413071	477777777413071	Yes
ERR688042	777377700003531	777377700003531	Yes
ERR983237	477777777413071	477777777413071	Yes
SRR1011506	677777477413771	677777477413771	Yes
SRR1011507	777777700003371	777777700003371	Yes
SRR1011511	677777477413771	677777477413771	Yes
SRR1011516	677777477413771	677777477413771	Yes
SRR1169470	477777777413071	477777777413071	Yes
SRR1169505	777777777413731	777777777413731	Yes
SRR1169527	407777777413071	407777777413071	Yes
SRR1169533	477777777413071	477777777413071	Yes
SRR1169569	477777777413071	477777777413071	Yes
SRR1169589	477777777413071	477777777413071	Yes
SRR1173692	777777777413731	777777777413731	Yes
SRR1510036	677777477413771	677777477413771	Yes
SRR1510037	677777477413771	677777477413771	Yes
SRR1510038	677777477413731	677777477413731	Yes
SRR1510039	677777477413771	677777477413771	Yes
SRR1510041	677777477413771	677777477413771	Yes
SRR1510067	677777477413771	677777477413771	Yes
SRR2086486	674000003413771	674000003413771	Yes
SRR2086487	674000003413771	674000003413771	Yes
SRR2086488	674000003413771	674000003413771	Yes
SRR2086489	674000003413771	674000003413771	Yes
